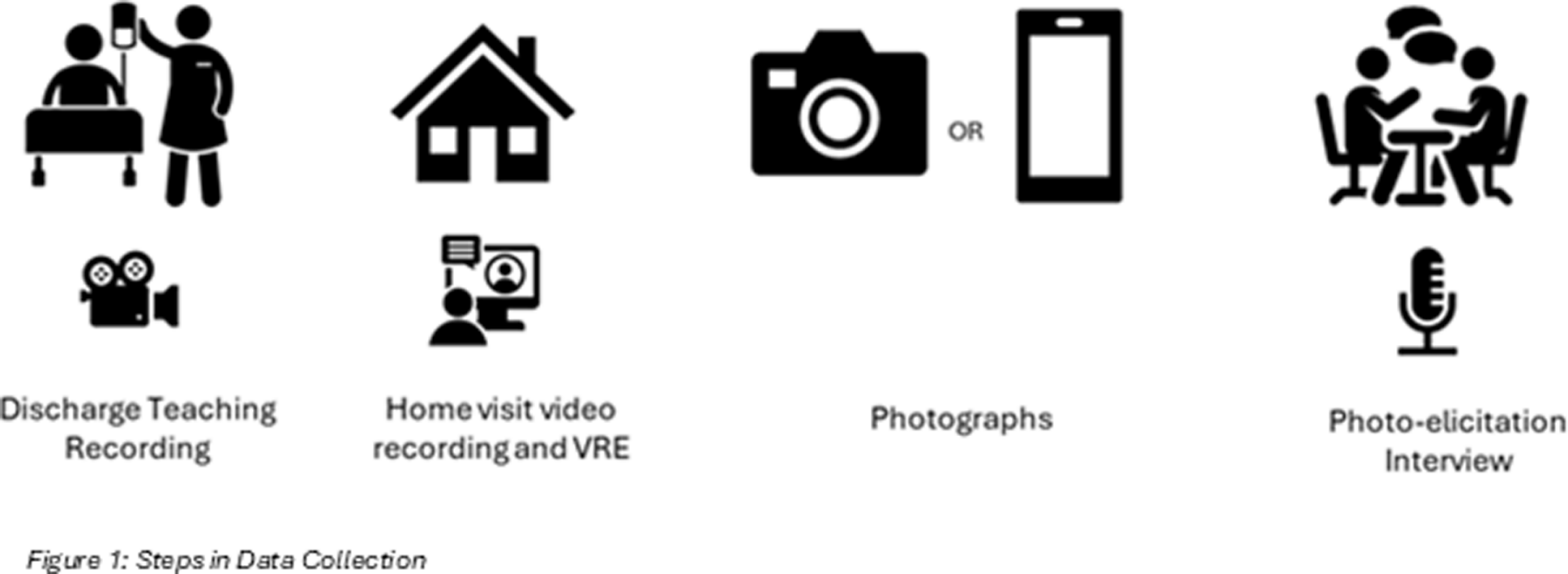# 368 Impact of a Multi-Modal Intervention Bundle on CLABSI Reduction across a Long-Term Acute Care Hospital System

**DOI:** 10.1017/ash.2026.10704

**Published:** 2026-06-23

**Authors:** Molly Harrod, Lauren Gauntlett, Allison Ranusch, Nicholas Henry, Danielle Helminski, Amanda Blok, Milisa Manojlovich, Elizabeth Scruggs-Wodkowski, Sara Keller, Manon Nitta, Sarah Krein

**Affiliations:** 1 VA Ann Arbor Healthcare System, Center for Clinical Management Research; 2 VA Ann Arbor Healthcare System; 3 Ann Arbor VA Hospital Medical Center; 4 Center for Clinical Management Research, VA Ann Arbor Healthcare System; 5 VA Center for Clinical Management Research; 6 University of Michigan; 7 Johns Hopkins University; 8 VA Ann Arbor Center for Clinical Management Research; 9 VA Ann Arbor Hlthcare System and University of Michigan

## Abstract

**Background:** Outpatient Parenteral Antimicrobial Therapy (OPAT) moves specialized, technically demanding care from hospitals to homes, where patients and caregivers take on tasks performed traditionally by trained nurses. However, infection prevention research rarely examines the home environment where OPAT administration and intravenous (IV) line management increasingly occur. Standard quantitative and qualitative approaches (e.g., surveys, interviews) may miss subtle medication administration deviations, environmental constraints, and other challenges that influence IV catheter- and medication-related risks. Video Reflexive Ethnography (VRE) and Photo Elicitation Interviews (PEI) offer a way to observe medication administration processes directly and to incorporate patient and caregiver perspectives to improve infection prevention education and practices. **Methods:** Using a VRE approach, we videorecorded discharge teaching and in-home OPAT routines, then conducted reflexive sessions in which patients and caregivers reviewed their own discharge teaching videos and discussed aspects of the process they found helpful, unclear, or challenging within their home environments. PEI added a participant-driven perspective by inviting patients and caregivers to photograph features of their home care setup that facilitated or hindered antibiotic administration and IV handling. Figure 1 demonstrates the steps in data collection. The Systems Engineering Initiative for Patient Safety **Results:** Observations of four patients, both in hospital and at home, revealed that visual methods captured behavioral details (e.g., using teeth to open syringe packaging) not typically discussed in interviews. Reflexive sessions and photo elicitation interviews highlighted uncertainties (e.g., doubts about correct processes), challenges with medication or IV-line maintenance (e.g., difficulty with IV clamps), and practical strategies for managing complex care tasks at home (e.g., hanging IV bag from wall hook rather than IV pole). These methods provided deeper insights into patients' experiences with care. **Conclusions:** This study demonstrates how VRE and PEI used together can strengthen infection prevention research. Both methodologies in concert provide new insight into how real-world patient and caregiver behaviors, constraints, and teaching gaps shape IV-line care, medication administration, and overall safety outside the hospital. By providing a clearer view of OPAT-related care as it occurs in home settings, this work shows promise for informing future refinement of education and IV medication administration guidance. This work is especially relevant given the growing complexity of infection-related care, including care delivered outside the hospital setting, requiring new approaches to understand and support patient safety.

**Figure:**